# Abetalipoproteinemia With a Novel Microsomal Triglyceride Transfer Protein Gene Mutation and Unique Oxidized Lipoprotein Levels

**DOI:** 10.14740/jmc5341

**Published:** 2026-09-04

**Authors:** Masato Hamasaki, Kazuo Hara, Kazuhiko Kotani

**Affiliations:** a Center for Community Medicine, Jichi Medical University, Shimotsuke, Tochigi 329-0498, Japan; b Division of Endocrinology and Metabolism, Jichi Medical University Saitama Medical Center, Omiya, Saitama 330-8503, Japan

**Keywords:** Cardiovascular risk, Low-density lipoprotein, High-density lipoprotein, Lipid disorders, Microsomal triglyceride transfer protein, Oxidative stress

## Abstract

Abetalipoproteinemia (ABL) is a rare autosomal recessive disorder caused by pathogenic variants in the microsomal triglyceride transfer protein (MTTP) gene, resulting in the defective secretion of apolipoprotein B (APOB)-containing lipoproteins. Although ABL can theoretically have a low risk of cardiovascular disease (CVD) due to the defective secretion of APOB-containing lipoproteins, the previously reported cases of ABL are limited and its CVD-related pathophysiology thus remains unestablished. We encountered a 25-year-old male patient with very low levels of lipids and lipoproteins. In addition to observing his signs and symptoms, we assayed serum oxidized forms of low-density lipoprotein (LDL) and high-density lipoprotein (HDL) particles using an enzyme-linked immunosorbent assay (ELISA). Variants in the APOB and MTTP genes were analyzed using next-generation sequencing (NGS). As a result, the patient exhibited low levels of total cholesterol (TC), triglycerides (TGs), LDL-cholesterol, and HDL-cholesterol. The oxidized LDL level was measured under a detectable limit, while oxidized HDL showed a relatively high level. An in-frame deletion in MTTP (p.Ser615del), a novel type of gene mutation, was identified in a homozygous manner. The patient had no obvious signs or symptoms of fat malabsorption or any neurological manifestations. In summary, we described a case of ABL with a novel MTTP mutation, p.Ser615del, without typical signs or symptoms. There were markedly low levels of atherogenic lipids and oxidized LDL, while the relatively high level of oxidized HDL with the low level of HDL-cholesterol appeared, which might offer unique insights into the pathophysiology of CVD-related risk in ABL. Further data accumulation of ABL is expected to clarify the disease picture.

## Introduction

Abetalipoproteinemia (ABL; OMIM #200100) is an autosomal recessive disease caused by a mutation in the microsomal triglyceride transfer protein (MTTP) gene [1]. MTTP is an essential molecule for the transfer of cholesterol esters and triglycerides (TGs) to apolipoprotein B (APOB)-containing lipoproteins. APOB is also crucial for the formation of chylomicron, very low-density lipoprotein (VLDL), and low-density lipoprotein (LDL). In patients with ABL, the secretion of APOB-containing lipoproteins is disordered, and gastrointestinal, neuromuscular, and ophthalmological symptoms due to the malabsorption of dietary fat and neurological manifestations due to deficiencies in fat-soluble vitamins are typically present [2]. ABL is a very rare disease, and about 100 cases with a total of 74 mutations have been reported in the literature [3]. Of these, five Japanese patients with six mutations have been previously reported [4–6]. Most mutations are known to be deletions in either the MTTP or binding regions of APOB or TG [7].

Because APOB-containing lipoproteins, mainly LDL, play a central role in atherosclerotic formation, patients with ABL are thought to have a low risk of cardiovascular disease (CVD) [6, 8]. The transport of circulating LDL particles to the arterial wall, along with their retention and modification, promotes atherosclerotic foam cell formation and plaque development [9]. The near absence of LDL in patients with ABL can theoretically confer protection against atherosclerosis. However, the CVD outcomes in patients with ABL have not completely been established in limited studies owing to the rarity of this disease [8]. More studies are required to understand the pathophysiology of atherosclerosis and CVD using multifaceted approaches, including additional CVD-related biomarkers.

Evaluating the oxidative modification of lipoproteins is a recent approach as such qualitative changes in lipoproteins have been linked to atherosclerosis [10]. Oxidized low-density lipoprotein (ox-LDL) plays an important role in atherogenesis, thereby promoting endothelial dysfunction, the macrophage uptake via scavenger receptors, and vascular inflammation [11]. Oxidized high-density lipoprotein (ox-HDL) can also indicate the impairment of anti-atherogenic properties of HDL particles through reverse cholesterol transport from the peripheral arteries to the liver tissues [12]. Measuring these oxidized lipoprotein species provides valuable information on the pathophysiology of CVD-related risk in ABL. We herein present a case of ABL with an MTTP mutation and oxidized lipoprotein levels.

## Case Report

We present a 25-year-old male patient with marked low levels of lipids, which was found in a blood test. The patient was referred to our hospital and there were no apparent signs and symptoms such as CVD, gastrointestinal, neuromuscular, ophthalmological, and neurological manifestations. Written informed consent was obtained from the patient for the further examinations and this case report. The study was approved by the Ethics Committee of Jichi Medical University.

After sampling the blood from the patient, the serum levels of total cholesterol (TC), TG, HDL-cholesterol (HDL-C), apolipoprotein A-I (apoA-I), and APOB were measured [13]. The level of LDL-cholesterol (LDL-C) was calculated using the Friedewald equation [14]. As markers of ox-LDL, serum amyloid A-LDL (SAA-LDL) and malondialdehyde-modified LDL (MDA-LDL) levels were measured using enzyme-linked immunosorbent assays (ELISA; Health Sciences West Japan, Japan for SAA-LDL and Mercodia Co. Ltd., Sweden for MDA-LDL). The ox-HDL level was also measured using an ELISA (Health Sciences West Japan, Japan) [12].

Genetic mutations in the APOB and MTTP genes were analyzed using next-generation sequencing (NGS), which was performed using 50 ng of genomic DNA with a NextSeq500 (Illumina Co. Ltd., USA). Library preparation was conducted using a TruSight One sequencing panel (Illumina Co. Ltd., USA). Variant data for the genes were obtained using the ANNOVAR tool [15], and the analyzed variants of amino acid substitutions and splice regions were interpreted using the ClinVar database [16]. Reference sequences were obtained from RefSeq: NM_000253.

Data of the patient are presented in Table 1. The measurement results of lipids identified the diagnosis of ABL; that is, very low levels of TC, LDL-C, and TG were observed. The level of HDL-C was also low. Similarly, a very low, undetectable level of APOB was observed, while the level of apoA-I was low. In addition, the SAA-LDL and MDA-LDL levels were very low, namely under or near the limit of detection, while ox-HDL showed a relatively high level. While there were no variants of APOB, an in-frame deletion in MTTP, p.Ser615del, in exon 13 was detected in a homozygous manner. This was found to be a novel type of gene mutation.

**Table 1:** Data of the patient

Measurements	Results	Reference range
Total cholesterol, mg/dL	35	140–220
HDL-cholesterol, mg/dL	25	40–80
Triglycerides, mg/dL	1	40–150
LDL-cholesterol, mg/dL	10	70–140
MDA-LDL, unit/L	0.8	7–110
SAA-LDL, µg/mL	0 (undetected)	15–50
Oxidized HDL, unit/mL	461	5–110
Apolipoprotein A-I, mg/dL	77	110–180
Apolipoprotein B, mg/mL	0 (undetected)	70–120
MTTP gene mutation	p.Ser615del/p.Ser615del	No mutation

HDL: high-density lipoprotein cholesterol; LDL: low-density lipoprotein; MDA-LDL: malondialdehyde-modified low-density lipoprotein; SAA-LDL: serum amyloid A-low-density lipoprotein; MTTP: microsomal triglyceride transfer protein

## Discussion

We herein present a case of ABL. An in-frame deletion in MTTP, p.Ser615del, was identified as a novel type of gene mutation in a homozygous manner. Very low levels of ox-LDL, and the relatively high level of ox-HDL were also newly revealed. Given the limited number of studies owing to the rarity of this disease [8] and the absence of studies on the measurement of oxidized lipoproteins, the information obtained from our patient may offer unique insights into the pathophysiological understanding of CVD-related risk in ABL.

Given the possible role of variants of MTTP gene in lipid profiles in ABL [1, 7], structural alteration at residue Ser615, located in the TG/cholesterol ester-binding pocket, can change the functions, resulting in the defective secretion of APOB-containing lipoproteins [17]. Ser615del is assumed to be a partially functional mutation because the in-frame deletion as seen in our patients is minimally affected rather than a large deletion [17]. It may explain that our patient retained the low level of HDL-C relative to the very low levels of LDL-C and TG. It might be also associated with the observation that our patient did not exhibit any malabsorption of dietary fat or neurological manifestations due to a lack of fat-soluble vitamins because HDL that was retained is able to be a carrier of the vitamins [17, 18].

The phenomenon of low level of HDL-C as in our patient has been reported with previous studies [4, 5, 19], and this can be discussed from various aspects of lipid metabolism. The low level of HDL is generally speculated to be due to an impaired HDL biogenesis resulting from the absence of APOB-containing lipoproteins [18], which are a source of HDL providing lipids for HDL maturation [18]. However, the production of HDL particles occurs throughout not only such pathways, but also other pathways of organs including renal and small intestine tissues [20, 21]. These complex metabolic conditions may be partly attributed to the level of HDL-C in ABL.

In our patient, SAA-LDL and MDA-LDL, markers of ox-LDL, were under or near detectable levels. Oxidized lipoproteins, such as ox-LDL, give a qualitative assessment of the atherogenic burden beyond the simple LDL-C quantification [10, 22]. As ox-LDL contributes to CVD [23], the very low level of ox-LDL observed in our patient may assist the evidence that patients with ABL have a low risk of CVD. Nonetheless, this is the single case study, and the ox-LDL level might be simply the consequence of absence of LDL and APOB. We must wait for further investigation of the relevance of ox-LDL in CVD-related risk.

On the other hand, there was the relatively high level of ox-HDL in our patient. This may reflect a partly impaired/delayed turnover of reverse cholesterol transport due to the remaining presence of HDL/apoA-I and the absence of LDL-C in peripheral arteries and the inactivity of lipid transfer. In addition, deficiency of vitamin E, an antioxidant, is commonly seen in ABL [24]. These factors might make HDL susceptible to oxidative modification [24]. In general, ox-HDL indicates the impairment of the anti-atherogenic properties of HDL particles, which can increase the CVD-related risk [12]. However, although the ox-HDL level appeared to be high relative to the levels of HDL-C and apoA-I, the ox-HDL level could show individual variability and a definitive threshold of ox-HDL level predicting CVD events has not been established [12]. As this level of ox-HDL does not fully guarantee the CVD-related risk, the ox-HDL in ABL merits future examination.

There are some limitations associated with this study. The serum level of vitamins (e.g., vitamins A and E) was not examined. In the genetic examination, we did not obtain any information on family members (i.e., co-segregation) or functional analysis of this mutation. Although no variants of APOB gene and no findings of acanthocytosis were detected in our patient, a wide range of presentations (e.g., fatty liver) and analyses of lipid- and lipoprotein-related genes (e.g., *PCSK9, ANGPTL3*) should be explored to differentiate more precisely from similar disorders such as familial hypobetalipoproteinemia. Such data would be addressed in future studies.

### Conclusions

In summary, a case of ABL was presented with a novel MTTP mutation, Ser615del, without typical signs or symptoms. The patient showed very low levels of atherogenic lipids, mainly LDL-C and ox-LDL, while the relatively high level of ox-HDL with the low level of HDL-C was observed. These findings might offer unique insights into the pathophysiological understanding of CVD-related risk in ABL. Therefore, further studies are warranted.

## Data Availability

Data will be made available upon reasonable and ethically-proper requests from the corresponding author.

## Abbreviations

ABL: abetalipoproteinemia
apoA-I: apolipoprotein A-I
APOB: apolipoprotein B
CVD: cardiovascular disease
ELISA: enzyme-linked immunosorbent assay
HDL: high-density lipoprotein
HDL-C: high-density lipoprotein cholesterol
VLDL: very low-density lipoprotein
LDL: low-density lipoprotein
LDL-C: low-density lipoprotein cholesterol
MDA-LDL: malondialdehyde-modified low-density lipoprotein
MTTP: microsomal triglyceride transfer protein
NGS: next-generation sequencing
OMIM: Online Mendelian Inheritance in Man
ox-HDL: oxidized high-density
ox-LDL: oxidized low-density lipoprotein
SAA-LDL: serum amyloid A-low-density lipoprotein
TC: total cholesterol
TGs: triglycerides
